# Editorial: Advancing sustainable management of fungal diseases in berry crops

**DOI:** 10.3389/fpls.2025.1612224

**Published:** 2025-05-16

**Authors:** Manuel Avilés, Peter Montgomery Henry, Berta De Los Santos, Angel Rebollar-Alviter, Celia Borrero

**Affiliations:** ^1^ Department of Agronomy, Universidad de Sevilla, Seville, Spain; ^2^ Agricultural Research Service, United States Department of Agriculture, Salinas, CA, United States; ^3^ Centro Las Torres-Tomejil, Instituto de Investigación y Formación Agraria y Pesquera, Junta de Andalucía, Alcalá del Río, Sevilla, Spain; ^4^ Centro Regional Morelia, Universidad Autónoma de Chapingo, Morelia, Michoacán, Mexico

**Keywords:** *Colletotrichum*, *Verticillium*, deep learning, *Phytophthora*, *Botrytis*

Berry crops, including strawberries, are economically important worldwide. However, their production is frequently threatened by a range of fungal and oomycete diseases, necessitating effective and sustainable management strategies. Conventional methods often rely on chemical pesticides, which raise concerns about environmental impact, human health, and the development of pathogen resistance. Consequently, there is a growing need for innovative and environmentally friendly approaches to protect these valuable crops. This editorial highlights recent research focused on advancing sustainable management of fungal diseases in berry cultivation, encompassing improved disease detection, understanding plant-pathogen interactions at the molecular level, and exploring alternative control methods.

Early and accurate disease detection is crucial for timely intervention and minimizing crop losses. Kim et al. addressed this challenge by proposing an improved vision-based detection method for strawberry diseases using deep learning. Their work demonstrated that a deep neural network (DNN) incorporating PlantNet, a backbone feature extractor pre-trained on plant data, and a two-stage cascade detection model could effectively identify strawberry diseases. This approach offers the potential for integration into automated robotic systems, enabling early detection and targeted interventions, thus reducing the reliance on broad-spectrum treatments.

Understanding the molecular interplay between the host plant and the pathogen is fundamental for developing targeted disease management strategies. Several recent studies have delved into these interactions in strawberry. Zhang et al. conducted a genome-wide analysis of the LysM gene family in octoploid strawberry and examined their expression in response to infection by *Colletotrichum fructicola*, a causal agent of anthracnose. LysM receptor-like kinases (LysM-RLKs) play crucial roles in plant immunity by recognizing chitin, a major component of fungal cell walls. Identifying and characterizing these genes in strawberry can provide insights into the plant’s defense mechanisms against fungal pathogens and potentially inform breeding strategies for enhanced resistance.

Focusing on another significant pathogen of strawberry, *Phytophthora cactorum*, Ghimire et al. performed a transcriptome analysis of the pathogen during infection of susceptible strawberry. Their research aimed to identify RXLR effectors, proteins secreted by oomycetes to manipulate host cells and promote infection. The identification of these effectors and understanding their function in inducing cell death contributes to our knowledge of *P. cactorum* pathogenesis and may reveal potential targets for disease control interventions.

Ozbudak et al. investigated the transcriptome analysis of the interaction between *Colletotrichum nymphaeae* and strawberry, another species within the anthracnose-causing *Colletotrichum acutatum* species complex. Their study identified in planta expressed genes of the fungal pathogen that are potentially associated with virulence. The significant expression of genes encoding enzymes like tannase, endochitinase, and chitinase during infection in different strawberry tissues (wounded leaves, unwounded leaves, and fruits) highlights the active role of these fungal factors in disease development. This knowledge can aid in understanding the infection process and identifying targets for disruption.

Beyond understanding the disease and the pathogen, developing sustainable control methods is paramount. Hernández-Muñiz et al. explored the optimization of anaerobic soil disinfestation (ASD) as a non-chemical alternative for controlling *Verticillium dahliae*, the causal agent of Verticillium wilt in strawberry. By using local agrowastes such as rice bran and residual strawberry extrudate as soil amendments under anaerobic conditions, they aimed to reduce the pathogen inoculum in the soil. Their findings indicated that rice bran amendment at an optimized rate effectively reduced *V. dahliae* density, demonstrating the potential of ASD utilizing locally available organic residues as a sustainable alternative to chemical fumigants.

While not directly focused on strawberry, the research by Chen et al. on resistance enhancement in tomato fruit against *Botrytis cinerea* using bacterial volatile organic compounds (VOCs) offers a glimpse into another promising avenue for sustainable disease management in horticultural crops. The induction of resistance through microbial VOCs could potentially be adapted for berry crops, providing an environmentally friendly approach to protect against postharvest and field diseases by activating the plant’s own defense mechanisms.

In conclusion, the studies highlighted here represent significant advancements in the sustainable management of fungal diseases in berry crops. From leveraging deep learning for early disease detection to dissecting the molecular interactions between strawberry and its pathogens, and exploring eco-friendly control methods like anaerobic soil disinfestation and induced resistance, these research efforts contribute valuable knowledge and innovative strategies for protecting berry production while minimizing environmental impact. Further research and development in these areas are crucial for ensuring the long-term sustainability and productivity of berry cultivation.

